# “Antibiotic prescribing etiquette” an elective course for medical students: could we recruit potential physicians to fight resistance?

**DOI:** 10.1186/s12909-022-03949-9

**Published:** 2023-01-05

**Authors:** Rehab H. El-sokkary, Shahenda G. Badran, Omnia S. El Seifi, Yara M. El-Fakharany, Rehab M. Elsaid Tash

**Affiliations:** 1 Medical Microbiology and Immunology Department, Faculty of Medicine, Zagazig University, Zagazig , Egypt; 2 Department of Family and Community Medicine, faculty of medicine, University of Tabuk, Tabuk, Kingdom of Saudi Arabia; 3 Community, Occupational and Environmental Medicine Department, Faculty of Medicine, Zagazig University, Zagazig, Egypt; 4 Forensic Medicine and Clinical Toxicology Department, faculty of medicine, Zagazig University, Zagazig , Egypt

**Keywords:** Medical school, Curriculum, Competencies, Antibiotic stewardship, Antibiotic resistance

## Abstract

**Background:**

A better understanding of medical students’ competencies about antimicrobial resistance and their use could facilitate a more effective education for them as future prescribers. The aim is to explore the educational impact of an elective course on medical students’ knowledge, perception, and attitude toward antibiotic resistance and use.

**Methods:**

Between December 2021 and January 2022, when a 2-credit hours elective course was designed and implemented, this interventional study was conducted. *The primary outcome measure* was the change in medical students’ knowledge, perception, and attitude about antibiotic resistance and use. Using a pre-post course questionnaire, this outcome was assessed. *The secondary measure* included students’ perception of the course; assessed by a post-course online survey.

**Results:**

Among the 50 enrolled students, the total knowledge score had significantly increased after the course with 95% CI After the course, with medium effect size ( Cohen’s d= -0.7 ) the participants’ mean ± SD total perception and attitude scores had significantly increased (52.38 ± 5.53 vs. 56.84 ± 5.86) respectively, (*p* = 0.000) with large effect size (( Cohen’s d= -0.8) There was a significant positive correlation between the total knowledge, attitude, and perception after the course (*r* = 0.542, *p* < 0.01). The mean ± SD of the overall course satisfaction was 4.20 ± 0.94. out of 5.

**Conclusion:**

Medical students’ knowledge, perceptions, and attitudes towards antimicrobial prescription have been improved after the elective antibiotic prescribing etiquette course. Elective courses could offer a great opportunity to enable the students to understand the extent of the problem, stand on the facts, and take responsibility for the antibiotic resistance crisis.

**Trial registration:**

NA

**Supplementary Information:**

The online version contains supplementary material available at 10.1186/s12909-022-03949-9.

## Background

Currently, antibiotics are routinely misused and overused, hastening the development of bacterial resistance and resulting in problems such as extended hospital stays, increased medical expenses, and death [1]. We are in an urgent need to modify the way antibiotics are prescribed and used [2]. Students’ education could lead to well-informed adults appreciating the risk of increased antibiotic resistance and raising concerns about the over-prescription of antibiotics to limit such practices [3, 4]. The situation is of more concern when it comes to medical students. As citizens and potential prescribers, they have a dual role in this crisis prevention[5, 6].

In our institution, as is the case in many other medical schools in low-middle income countries, antibiotic use knowledge is developed during medical training, including lectures on the basics of antibiotic treatment and textbooks on infectious diseases. However, many physicians continue to prescribe antibiotics inappropriately [7]. This can be owed to the extent to which the problem is realized within the facility and its incorporation into the routine practice, and leadership support, [8,9].

From an educational perspective, the current teaching approaches and textbooks do not adequately prepare medical students for this task [9–11]. Many limitations do exist (i) the learning process is passive and students are not encouraged to develop an adequate level of critical thinking [12]; (ii) students memorize theoretical medical concepts, rather than learning to solve the practical problems that arise in medical practice; (iii) students focus on clinical situations encountered in hospitals, but not in primary care; (iv) students focus on a single patient, whereas, in real life, they often have to manage several patients simultaneously.

Many researchers. investigated the knowledge, attitude, and practice toward antibiotic use among medical students [9, 13–15]. Many teaching opportunities were designated juniors as problem-based learning, role-play games, round tables, musicals as well as e-learning [10].

In the current curriculum, the delivery of many competencies about the rational use of antibiotics is missed. It is taught in the form of separate topics in microbiology, pharmacology, public health, and clinical sciences. No separate theme for antimicrobial resistance exists in the current curricula. Comprehensive evaluations of antimicrobial stewardship teaching programs and overall effectiveness in undergraduate medical institutions are still few and sparse [16, 17]. As far as we know, not enough data are available about the impact of antibiotic use in educational courses in middle-income countries like Egypt. Given the good educational impact of elective courses on students’ learning [18], we designed an elective course titled “antibiotic prescribing etiquette” to help medical students better understand their role in combating the antimicrobial resistance crisis. We hypothesized that this course could improve the comprehension of medical students about their role in the proper antibiotic prescription process. In this study, we aimed to explore the educational impact of this course on medical students’ knowledge, perception, and attitude toward antibiotic resistance as well as proper antibiotic use and prescription. We anticipated the findings may help curriculum designers to consider adding this topic to the main curriculum.

## Methods

This intervention prospective study was conducted in the Faculty of Medicine, Zagazig University; a leading medical school in Egypt from December 2021 to January 2022.

### **Study population**

We used a purposive sample in this study that included all the enrolled students in this elective course (50 students) with a 100% response rate. Informed written consent was collected from each student.

The enrolment of medical students: This elective course is delivered to third-year medical students. The course announcement and communication occurred via the official online platform; Microsoft teams.

At the time of course enrollment, our students have already studied microbiology, pharmacology, introduction to clinical practice, and infection control courses.

### Study intervention “course description”

“Antibiotic prescribing etiquette” is a 2-credit hours elective course, that was delivered over 2-weeks duration. It was designed and prepared by experts in antimicrobial resistance, infection control, and medical education, this followed the methodology mentioned earlier for course design [10, 19–21]. First were revised earlier published reports about the selected topic [9, 13–15], revised the current curriculum, revised the national academic standards for medical students (NARS) 2017 [22], and the final course reports delivered from all related modules that were given in our faculty and are related to the antibiotic resistance topic. The course was designed with the aim to improve the awareness of medical students about antimicrobial resistance and identify how they could apply the proper prescription rules. The course specification was revised by the official MBBCh curriculum committee to revise the objectives’ alignment with the teaching methods to achieve the course goals, check the course quality and approve the suitability to teach the course as an elective course.

Learning outcomes and teaching methods: Course directors defined a list of learning outcomes (Los) that conveys what students would be able to do after completing the course, they were distributed upon the 3 learning domains: knowledge, attitude, and skills, Table 1.

**Table 1:** The course matrix showing the learning outcomes, teaching methods, and types of sessions

**Activities/teaching methods**	**Learning outcomes (Los)**									
	Knowledge domain^a^			Attitude domain^a^			Skills domain^a^			
	To relate the previously acquired information to real life situations	To compare The appropriate antibiotic prescription to the fault practices	To conclude, the role of antibiotic prescription is to prevent antibiotic resistance	To suggest suitable actions to prevent the spread of antibiotic resistance	To appraise the role of the key players in the antibiotic use	To investigate their role in the antibiotic resistance problem	To argue their viewpoint in respectable manner	To persuade the audience with their message	To create a well-structured message that matches the target group	To assemble the appropriate components of audiovisual aids to deliver their message
Workshop	+	+	+							
Case scenario	+	+	+				+	+	+	+
Cartoon animations	+	+	+							
Quotes from movies	+	+	+							
Real life stories	+	+	+							
Self-reflection	+	+	+	+	+	+				
Lecturette	+	+	+			+				
Face-to-face discussion	+	+	+	+	+	+	+	+	+	+
Online discussion	+	+	+	+	+	+	+	+	+	+

^a^Session type for knowledge and attitude domains were interactive sessions, lectures, and reflective performance assignment. For skills domain interactive sessions and the reflective performance assignment were used

We considered making the learning process attractive, exciting, and challenging using different teaching and learning methods [20]. This included 2 interactive sessions: an online and a face-to- face one, and 2 recorded lectures, and a reflective practice assignment. The latter is a health education message directed to one of the key players involved in the antibiotic prescription process. The students were requested to design and record a 2-minute multimedia file of their message.

**Fig. 1:**
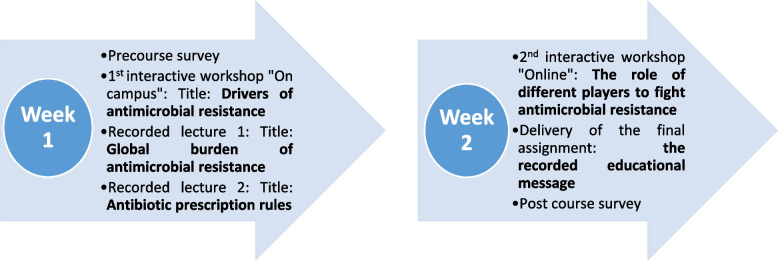
Course map

Table 1 presents the course Los, the teaching activities, and the type of sessions in the three domains: knowledge, attitude, and skills. Course flow is illustrated in Fig. 1.

### Learning environment

The course was developed for use in a class of 30–50 students, which was then divided into groups of three to five students for a small-group activity and the submission of the final assignment. We presented the session in a classroom equipped with a computer and projector, and students received handouts to guide them through the small-group work. The online session and the recorded lectures were conducted via the educational platform; Microsoft teams.

Criteria to pass the course included attendance, active participation, and the health education message. The latter was evaluated and scored according to a constructed checklist. The best 3 assignments were awarded for their creativity.

*Session type for knowledge and attitude domains were interactive sessions, lectures, and reflective performance assignment. For skills domain interactive sessions and the reflective performance assignment were used.

### Outcome measures

*The primary measure* was the change in the medical students’ knowledge, perception, and attitude about antibiotic resistance and use. This was measured by a pre-post course questionnaire that was designed after reviewing literature [9, 19, 23, 24], revised, piloted, and validated.

The content validity of the questionnaire was assessed in terms of necessity and relevance, by 10 professional experts in the field. The necessity of questions was scored on a 3-point scale from (1–3) " not necessary, useful but not necessary, necessary” respectively. The relevance of the items was assessed on a scale of 1 to 4 ; [1: not relevant- 2: somewhat relevant and needs some revision, 3: quite relevant, 4: highly relevant]. Both the Content Validity Ratio (CVR) and the Scale Content Validity Index (S-CVI) for the questionnaire were calculated to be acceptable values of 0.83 and 0.87, respectively [25, 26].

It was divided into 4 sections: (1) demographics including age, gender, and motive to join the course. (2) prior experience in antibiotic use, as well as the source of information. (3) knowledge-related items. (4) perceptions and attitude. The post-course questionnaire included the same items mentioned in the pre-course survey in the knowledge section, as well as in the perception and attitude section.

*The secondary measure* included students’ perception of the elective course. At the end of the course, the students were requested to fill in an online survey form [10, 19]. This encompassed their interest in the course (3 items), as well as their satisfaction with the course design and implementation (7 items). Open-ended questions (3 items) were included: what they liked best, the most they disliked, and a suggestion for improvement.

### Statistical analysis

Data were summarized and analyzed using SPSS 23 by calculating frequency, percentage, mean, and SD. Paired t-test and Wilcoxon Sign Ranks test were computed for the paired continuous data and McNemar’s test was for the paired nominal data to find the difference between the pretest and posttest results. Pearson’s correlation coefficient was calculated to determine the correlation between students’ satisfaction and the total knowledge,attitude, and perception scores. The significant level was determined as *p* < 0.05.

The knowledge section, and the perception and attitude section each composed of 22 items. The total score ranged from 22 to 66. The score was calculated on a 3-point Likert scale: Agree, gets 3 points, neutral 2 points and disagree 1 point. Ten items were calculated by reverse coding. This was applied to 3 items in the knowledge section, and 7 items in the attitude and perception section.

The effect size of our educational intervention on total knowledge, perception and attitude was assessed using Cohen’s term d by taking the difference between the means in the pretest and posttest for the means and divided it by the standard deviation of the pretest for both domains.

## Results

The course was delivered to 50 3rd year medical students: (26, 52%) males, and (24, 48%) females. Their mean age was 20.36 ± 0.63 years. When asked about their motive for joining the course, (35, 70%) were interested in the topic, (12, 24%) were attracted to the course title, and (3, 6%) joined because it was the only available course for them at the time of registration. Students’ personal experience in antibiotic use is summarized in Table 2.

The useful sources to get information about antibiotic use and resistance were arranged in descending order as follows: Formal lectures (84%), textbooks (78%), new technology such as the internet, smartphone applicants, etc. (72%), medical journals (58%) and social media (58%).

**Table 2:** Self-reported experience with antibiotic use among the study participants:

variable	category	No. (Total 50)	100.0%
Last time administering antibiotics	In the last year	19	38.0
	In the last six months	16	32.0
	In the last month	15	30.0
Obtaining antibiotics through the following:	A medical prescription by a physician	35	70.0
	Without a prescription from a pharmacy	8	16.0
	Leftovers from a previous course	5	10.0
	Recommended by friends/family	11	22.0
Indication of last antibiotic administration	Cough	2	4.0
	Cold/flu	19	38.0
	Fever	7	14.0
	Other respiratory problems	4	8.0
	Wound infection	5	10.0
	Ear/eye infection	2	4.0
	Diarrhea and other gastrointestinal problems	6	12.0
	Suspected/confirmed COVID-19	3	6.0
	Other infections	2	4.0

The total knowledge score of the participants has significantly increased after the course compared to the pre-course score; 48.06 ± 6.12 and 52.35 ± 5.91 respectively, *p* = 0.000. Table 3 shows the detailed knowledge items. The total perception and attitude scores of the participants significantly increased after the course (52.38 ± 5.53 & 56.84 ± 5.86) respectively, the mean difference was - 4.29 ± 5.04 with 95% CI (-5.75 to -2.82) (*p* = 0.000). Table 4 shows the detailed perception and attitude items.

The effect size of our educational intervention on total knowledge, perception and attitude equals - 0.7 for total knowledge which is considered as medium effect size and - 0.8 for total attitude and perception, that is a large effect size.

**Table 3:** Medical students’ knowledge about antibiotic resistance and use before and after the course (Mean ± SD)

Items	Categories	Before Mean ± SD	After Mean ± SD	P
How much do the following terms look familiar to you:	MRSA	2.48 ± 0.73	2.96 ± 0.19	0.000
	VRSA	2.18 ± 0.84	2.60 ± 0.70	0.010
	ESBL	1.48 ± 0.76	1.96 ± 0.72	0.001*
	MDR bacteria	2.58 ± 0.67	2.86 ± 0.40	0.001
	XDR bacteria	1.78 ± 0.88	2.18 ± 0.71	0.013*
	Pan drug resistance	1.90 ± 0.86	2.50 ± 0.73	0.001*
Antibiotic resistance is a worldwide public health problem		2.64 ± 0.77	2.84 ± 0.54	0.096
Antibiotic resistance is a nationwide public health problem		1.32 ± 0.74	1.32 ± 0.74	1.000*
Antibiotic resistance is an important and serious problem in local hospitals		1.64 ± 0.94	1.68 ± 0.95	0.796*
Antibiotic resistance is a problem that can be easily controlled		1.24 ± 0.65	1.16 ± 0.54	0.414*
Antibiotic resistance is an overrated problem with no serious impact		1.12 ± 0.47	1.08 ± 0.39	0.655*
Antibiotic use in self-limiting infections contributes to antibiotic resistance		2.56 ± 0.67	2.58 ± 0.70	0.875
Antibiotics for a duration shorter than that indicated contribute to antibiotic resistance		2.50 ± 0.70	2.70 ± 0.61	0.067
Antibiotics for a duration longer than that indicated contribute to antibiotic resistance		2.24 ± 0.82	2.48 ± 0.73	0.073*
Empiric antibiotic therapy contributes to antibiotic resistance		2.38 ± 0.60	2.46 ± 0.70	0.522
Lack of control in the sale of antibiotics in pharmacies contributes to antibiotic resistance		2.82 ± 0.52	2.74 ± 0.63	0.438
Self-medication is one of the primary causes of antibiotic resistance		2.68 ± 0.65	2.72 ± 0.64	0.642
Poor infection control measures contribute to antibiotic resistance		2.60 ± 0.67	2.76 ± 0.51	0.132
Excessive antibiotic use in livestock (animals reared for food) contributes to antibiotic resistance		2.36 ± 0.85	2.82 ± 0.52	0.001*
The use of antibiotics with a broader than necessary spectrum is one of the reasons for the development of antibiotic resistance		2.60 ± 0.69	2.66 ± 0.71	0.627
There are bacterial infections resistant to all available antibiotics		2.36 ± 0.82	2.72 ± 0.67	0.009*
Bacteria can transmit resistance genes between different species		2.72 ± 0.60	2.78 ± 0.41	0.569
**Total knowledge score**		**48.06 ± 6.12**	**52.35 ± 5.91**	**0.000**

*ESBL* Extended spectrum B lactamases, *MDR* multidrug resistant, *MRSA* methicillin resistant staphylococcus aureus, *VRSA* vancomycin resistant staphylococcus aureus, *XDR* extended spectrum B-lactamases. The paired t test was computed.

*Wilcoxon Sign Ranks test was computed

*p* <0.05 is significant

**Table 4:** Medical students’ perception and attitude toward antibiotic resistance and use before and after the course (Mean ± SD)

Categories	Items	Pretest Mean ± SD	Posttest Mean ± SD	P
Do you think you are sufficiently prepared to do the following after graduation?	Realize when to start antibiotic therapy	2.86 ± 0.45	2.88 ± 0.38	0.821
	Select the best antibiotic for each specific infection	2.52 ± 0.64	2.68 ± 0.47	0.159
	Correlate the basic mechanisms of antibiotic resistance in clinical context	2.52 ± 0.64	2.80 ± 0.40	0.007
	Able of interpreting antibiograms	2.14 ± 0.75	2.48 ± 0.57	0.010*
	Able to get reliable sources of information to treat infections	2.34 ± 0.87	2.66 ± 0.59	0.029*
	Realize why we switched from intravenous antibiotics to oral antimicrobials	2.52 ± 0.67	2.54 ± 0.61	0.875
Proper antibiotic prescription:	Patient's request of antibiotic is an indication for its prescription	2.44 ± 0.83	2.44 ± 0.83	1.000*
	Antibiotics’ sale without prescription should be prohibited	2.66 ± 0.59	2.82 ± 0.43	0.088
	In the presence of a cough and a sore throat, antibiotics are my first-choice treatment	2.60 ± 0.75	2.30 ± 0.86	0.054*
	I stop taking antibiotics when I feel better or when symptoms disappear	2.64 ± 0.66	2.28 ± 0.90	0.008*
	I usually know when I need antibiotics	1.94 ± 0.84	1.90 ± 0.76	0.802*
Proper antibiotic use	Missing one or two doses of an antibiotic treatment does not contribute to antibiotic resistance	2.44 ± 0.78	2.28 ± 0.75	0.226*
	Antibiotics are safe, so they could be commonly used	2.76 ± 0.51	1.56 ± 0.81	0.000*
Role as health care promoter	I can describe the role of each profession in appropriate antibiotic use	2.16 ± 0.84	2.52 ± 0.67	0.022*
	I can communicate about antibiotic use in a manner that engages the members of health team	2.24 ± 0.84	2.64 ± 0.63	0.013*
	I can describe collaborative approaches to appropriate antibiotic use	2.02 ± 0.79	2.30 ± 0.73	0.049*
	Training or education about antibiotics and resistance is very important for medical students	2.70 ± 0.58	2.86 ± 0.49	0.132
	When someone self-medicates with antibiotics, I try to persuade him/her not to do so	2.76 ± 0.55	2.86 ± 0.45	0.341
	I have informed family and friends about the risks associated with the use of non-prescribed antibiotics	2.68 ± 0.68	2.72 ± 0.67	0.687
Resistance preventive measures	I am confident that the development of new and effective drugs will keep pace with the growing rate of antibiotic resistance	2.10 ± 0.76	1.94 ± 0.73	0.240*
	I have taken infection control measures to prevent antibiotic resistance	2.56 ± 0.64	2.60 ± 0.72	0.735
	I adopt more precautions when using antibiotics after learning about antibiotic resistance	2.80 ± 0.53	2.76 ± 0.55	0.719
**Total score**		**52.38 ± 5.53**	**56.84 ± 5.86**	**0.000**

The paired t test was computed. *Wilcoxon Sign Ranks test was computed

*P* <0.05 is significant

After the course, we reported a significant positive correlation between the total knowledge score and the attitude and perception score (*r* = 0.542, *p* < 0.01).

### Perception of the students about their educational experience through the course

Out of the 50 students, 54% believed that it should be part of the main curriculum, most of them would go for an advanced course on the same topic (90%) and 94% would recommend the course to colleagues. The course met the expectations of 62% of the students. About 30% of the students recommended adding more details about antibiotic uses and types in cooperation with the pharmacology department. Following, more interactive face-to-face sessions were suggested by (14%), and be an obligatory module by (8%).

The overall satisfaction with the course was 4.20 ± 0.94. out of 5. A positive significant correlation was present between students’ satisfaction and total attitude and perception in post-test (*r *= 0.300, *p* < 0.05).

## Discussion

Improving prescribers’ education has lately been advocated to ensure safer prescribing and optimum antibiotic use [27]. We aimed to study the impact of an elective educational course on prescription-related competencies among undergraduate medical students. The study disclosed the statistically significant increase in the medical students’ knowledge, attitude, and perception scores after attending the course. The majority of the students were interested and satisfied with the course.

First of all, we owe the appropriateness of this course mainly to the fact that students attended electively without being bothered by exams or any stressful evaluation; most of them (94%) joined either because they were attracted to or were interested in the topic. Medical students perceive electives as a valuable experience, allowing them to tailor learning skills and qualifying them to early differentiate during medical training [28].

The course improved the medical students’ knowledge with medium effect size of -0.7. It added information regarding some common terminology in antimicrobial resistance; extended spectrum B lactamases (ESBL), extended resistant (XDR) bacteria, and pan drug-resistant bacteria. Those 2 terms, in specific, were addressed in the interactive sessions ; storytelling, and case scenarios. As previously reported [29–34], the course enhanced their awareness that antimicrobial resistance is a national public health threat with a serious impact that exists in their local hospitals as well.

Deficiencies in the medical school curricula, including fundamental concepts of antimicrobial usage have been reported in studies conducted in different countries such as the USA, China, Colombia, Singapore, and Italy [9]. In the current report, the course succeeded to bring attention that many drivers can lead to antimicrobial resistance. Unlike how the antibiotic resistance topic is taught in the main curriculum, in this course it was delivered as a single theme that covered both medical and non-medical contributing factors. In addition, as previously recommended [10], we used many activities to address knowledge deficiency. We believe that this helped to some extent in filling the students’ knowledge gaps.

This study showed significant improvement in the perception and attitude scores with large effect size of -0.8. After the course, students thought they are sufficiently prepared to interpret antibiograms and get the treatment information needed from reliable sources. Published reports emphasize how crucial it is to educate physicians about antibiograms. Though important, this point is not addressed in the undergraduate medical curriculum in our institution.

Students showed a significant improvement in their perception of proper antibiotic prescription indications, dosage, and duration. Previous reports recommended the delivery of properly designed educational courses targeting antibiotic use specific items in the three learning domains [35]. Not a small percentage of students in our study believed that improvement of symptoms is an indication for stopping the antibiotic course, which was considerably alarming. Following the course, a statistically significant shift in their perception was noticed. In concordance with this, Okedo-Alex S et al., [36] reported that 22% of the respondents stopped the course of antibiotics when they felt better. Increasing knowledge and improving the perception and attitude from the pretest to post-test with medium and large effect size respectively can be considered as an indicator of thevaluable impact of our work. .

Thus this elective course could be recommended for wider scale application ; moreover, it could be adapted to the obligatory courses for undergraduate students keeping the positive points feedback and opportunities for improvement in mind for future implementation rounds.

The course helped students to recognize and promote the role of different health team members in proper antibiotic use, from that notion came the idea of the assignment. We thought that delivering messages to targeted groups would help students understand the concept of an interprofessional environment, in which doctors and others work within complex clinical groups promoting collaborative practice among various professionals. Teaching others gives information retention by 90% [37]. Using videos added more dynamism; it not only gave them the opportunity to integrate, but also ensured the active participation of each student.

Ortega-Paredes et al., 2022 reported, about 60% agreed with the development of novel antibiotics as a good solution for the problem [38]. After the course, the students showed more confidence that the development of new and effective drugs will not keep pace with the growing rate of antibiotic resistance and hence this increased their awareness of the preventive methods of the antibiotic resistance problem.

Earlier reports [24, 39] recommended medical schools should devote more effort to teaching proper antibiotic usage. More than half of the respondents thought that the course should be included within the main curriculum and almost all would recommend the course to colleagues. The majority of the students were satisfied with the course, their satisfaction positively correlated with their perception and attitude. This was evident in their interest to join an advanced course on the same topic (90%).

### Limitation of the study

Many limitations existed that could affect the presented results. The course was of short length; a longer duration may give better results. Lacking a control group was a limiting factor. Although, this type of study design is accepted to be used in case of evaluation of a training program/course where the group is selected in a non-random way, and the randomization is impractical, however, it has many potential consequences, for instance, conveying the improved post-test results to other outside influencers. In the current study, the main outcomes were the knowledge and attitudes of students. To what extent medical students would translate these beliefs into effects in future clinical practice was not addressed. Further research is needed to tackle this domain.

## Conclusion

Our findings suggest that medical students’ knowledge, perceptions, and attitudes towards antimicrobial prescription have been improved after the elective antibiotic prescribing etiquette course.

This study affirmed the potentials elective courses could offer in the medical school curriculum. The investigated course provided a great opportunity to enable the students to understand the extent of the problem, stand on the facts, and take responsibility towards the antibiotic resistance crisis. However, further improvements to the course content may be considered in future research to include how to deal with patient expectations, how to manage specific types of infections, etc.

## Supplementary Information

**Additional file 1. **Data collected from the participants before the course

**Additional file 2.** Data collected from the participants after the course

## Data Availability

All data generated or analysed during this study are included in this published article (Supplementary file).
